# Supplementary material for: The causal role of circulating inflammatory markers in osteoporosis: a bidirectional Mendelian randomized study
Source: Front Immunol. 2024 Jul 18;15:1412298. doi: 10.3389/fimmu.2024.1412298 (PMC11291241; doi:10.3389/fimmu.2024.1412298)
Supplement: Supplementary file 1 [file DataSheet_1.zip › supplementary material/CIM-OSTEOPOROSIS /GCST90274809.csv_leave_one_out.pdf]

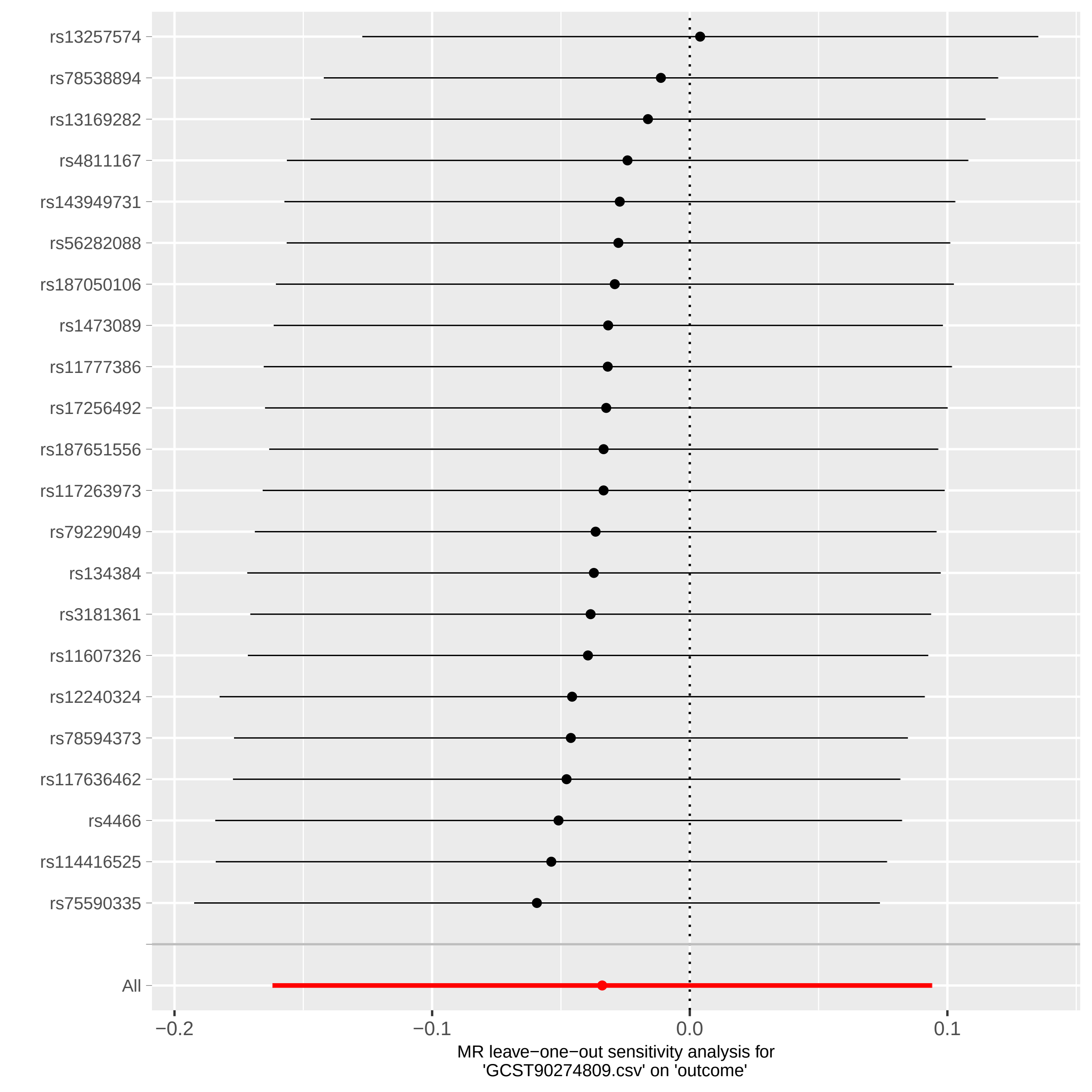
rs13257574
rs78538894
rs13169282
rs4811167
rs143949731
rs56282088
rs187050106
rs1473089
rs11777386
rs17256492
rs187651556
rs117263973
rs79229049
rs134384
rs3181361
rs11607326
rs12240324
rs78594373
rs117636462
rs4466
rs114416525
rs75590335
All
−0.2
−0.1
0.0
0.1
MR leave−one−out sensitivity analysis for
'GCST90274809.csv' on 'outcome'
